# What matters most? Assessment of within-canopy factors influencing the needle microbiome of the model conifer, *Pinus radiata*

**DOI:** 10.1186/s40793-023-00507-8

**Published:** 2023-05-30

**Authors:** Sarah Addison, Charlotte Armstrong, Kathryn Wigley, Robin Hartley, Steven Wakelin

**Affiliations:** 1 Scion, Private Bag 3020, Rotorua, 3046 New Zealand; 2 Scion, P.O. Box 29237, Riccarton, Christchurch, 8440 New Zealand

**Keywords:** Conifer, Phyllosphere, Community assembly, Model system, Microbiome

## Abstract

The assembly and function of the phyllosphere microbiome is important to the overall fitness of plants and, thereby, the ecosystems they inhabit. Presently, model systems for tree phyllosphere microbiome studies are lacking, yet forests resilient to pests, diseases, and climate change are important to support a myriad of ecosystem services impacting from local to global levels. In this study, we extend the development of model microbiome systems for trees species, particularly coniferous gymnosperms, by undertaking a structured approach assessing the phyllosphere microbiome of *Pinus radiata*. Canopy sampling height was the single most important factor influencing both alpha- and beta-diversity of bacterial and fungal communities (p < 0.005). Bacterial and fungal phyllosphere microbiome richness was lowest in samples from the top of the canopy, subsequently increasing in the middle and then bottom canopy samples. These differences maybe driven by either by (1) exchange of microbiomes with the forest floor and soil with the lower foliage, (2) strong ecological filtering in the upper canopy via environmental exposure (e.g., UV), (3) canopy density, (4) or combinations of factors. Most taxa present in the top canopy were also present lower in tree; as such, sampling strategies focussing on lower canopy sampling should provide good overall phyllosphere microbiome coverage for the tree. The dominant phyllosphere bacteria were Alpha-proteobacteria (Rhizobiales and *Sphingomonas*) along with Acidobacteria Gp1. However, the *P. radiata* phyllosphere microbiome samples were fungal dominated. From the top canopy samples, Arthoniomycetes and Dothideomycetes were highly represented, with abundances of Arthoniomycetes then reducing in lower canopy samples whilst abundances of Ascomycota increased. The most abundant fungal taxa were *Phaeococcomyces* (14.4% of total reads) and *Phaeotheca* spp. (10.38%). A second-order effect of canopy sampling direction was evident in bacterial community composition (p = 0.01); these directional influences were not evident for fungal communities. However, sterilisation of needles did impact fungal community composition (p = 0.025), indicating potential for community differences in the endosphere versus leaf surface compartments. Needle age was only important in relation to bacterial communities, but was canopy height dependant (interaction p = 0.008). By building an understanding of the primary and secondary factors related to intra-canopy phyllosphere microbiome variation, we provide a sampling framework to either explicitly minimise or capture variation in needle collection to enable ongoing ecological studies targeted at inter-canopy or other experimental levels.

**Supplementary Information:**

The online version contains supplementary material available at 10.1186/s40793-023-00507-8.

## Introduction

Microorganisms growing on and in plant leaves, i.e., the phyllosphere, can fundamentally influence host physiology and fitness [1–3]. An increasing body of evidence shows that microbiome-host interactions are expressed across a multitude of traits, from nutrient acquisition, disease resistance, drought tolerance, production of plant hormones, and even affecting exchange of gas and water between the plant and atmosphere [1, 4, 5]. In many cases, these associations are fundamental to the health and fitness of plants [6, 7] and have a strong co-evolutionary background [8–10]. Indeed, our fundamental perspective of microbiome associations is shifting from one of a ‘host tissue partnered with a microbial consortium’, to that of a ‘coalescence of plant and microbes existing and functioning as a single ecological entity’ [11].

It is clear the processes affecting assembly of the phyllosphere microbiome are important to overall fitness of plants and, therefore, the ecosystems they inhabit. Given the importance of the phyllosphere microbiome to plant fitness, it is not surprising that ecological filters operate on leaf microbiome assembly, favoring establishment of some taxa whilst suppressing others [12]. These filters are overlaid with – and operate in conjunction with - plant genetic factors that aid selection of the microbiome, and then maintain community structure over time [8, 13–15]. The occurrence of these is likely necessary due to leaves being in such an open environment and naturally subject to constant immigration of microbes; to maintain a stable and functioning microbiome, a variety of effective selection mechanisms must be expressed. This is supported by observations of convergence in microbiome composition over time, indicating a selection-based successional trajectory to be active [12]. Indeed, of the plant microbiome compartments assessed, spanning soil to leaves, endosphere to tissue surfaces, the leaf endosphere was found to be under the strongest host-based selective influence [16].

Soil is a common environmental source of microorganisms present in the phyllosphere. Transport from the soil to the plant typically requires atmospheric movement from wind eddies and uplift, however vectors such as invertebrates can also be important [16–18]. The other key source of microbial immigration is atmospheric deposition, particularly via precipitation (fog, rain) and dust [19–22]. As such, it is likely that for tall plants such as trees, that vertical distance from the ground has a role in affecting species immigrating onto the phyllosphere. This would apply especially to those taxa where their dispersion is limited by factors such as wind. Phyllosphere microbiomes that initially reflect soil-originated communities would have higher abundance in lower portions, with those reflecting atmospheric or other sources would have higher abundance in the upper reaches. Furthermore, cardinal direction may influence recruitment and selection via factors associated with, for example, prevalent wind direction and differential exposure to sunlight and rainfall. In these instances, impacts of tree form and canopy structure may also be expressed. In particular, leaves on the periphery (edges and top) of the canopy have stronger potential exposure to external sources of microbiomes, but also strong environmental selective pressures from conditions such as UV, wind flow, desiccation and wetting, frost, temperature fluctuations and so forth. Canopy edges can also be the primary area for new leaf growth, exposing new plant tissue for microbial recruitment. These factors are rarely assessed in plant microbiome research.

While the ecology of the phyllosphere microbiome is being explored on an increasing number of plant species, the vast majority of our understanding has been centred towards model species such as *Arabidopsis thaliana*, or commodity species such as rice, soybean, and maize [6, 23]. This is understandable given the opportunities to improve food crops and increase sustainability of agroecosystems. Yet, a third of Earth’s habitable land area is forested [24], representing key biomes such as temperate and tropical rainforests through to boreal ecosystems and savannahs. While the research into tree and forest microbiomes is advancing [25], the level of investment in resources is lamentable given the extent of these habitats and the magnitude of ecosystem services they support globally [26].

Trees and forest ecosystems are at considerable risk of ecological impact brought about by climate change. These systems are sessile and long-lived; they can neither migrate nor evolve at rates accordant with the pace of shifting abiotic (climate breakdown) and biotic (pests and diseases) stress [27]. Many of these biomes, such as the Amazon rainforest, are irreplicable, comprising unique biodiversity. However, the forest regions identified at most risk also include the massive northern latitude boreal forests spanning from Canada to Russia [28, 29], comprising ~ 30% of global forests. These are typically dominated by conifers (gymnosperms) including spruce (*Picea*), fir (*Abies*), pine (*Pinus*), and larch (*Larix*), along with angiosperm species such as birch (*Betula*), poplar and aspen (*Populus*) [28]. Model tree-microbiome systems are needed to develop our understanding of tree health, forest health, and ensuring resilience of delivery of forest ecosystem services, from savannahs to boreal regions. Models are needed for purposes spanning climate protection through to improved supply of forest products such as wood. These model systems must also include gymnosperm trees. These are largely lacking.

Monterey or radiata pine (*Pinus radiata* D. Don) provides a remarkable model species for assessing the gymnosperm microbiome and therein holobiome interactions and fitness. It is native to coastal California (Año Nuevo-Swanson, Monterey, Carmel, and Cambria) and Mexico (Guadalupe and Cedros Islands) [30]. As such, disjunct modern populations occur both onshore and on offshore islands, but fossil records indicate that Monterey pine was previously widely distributed [30, 31]. The species is on the IUCN Red List of Threatened Species [32], thus the preservation of the wild populations is important for both their intrinsic conservation value, but also as a source of wide genetic diversity for utilisation.

Out of its native range, *P. radiata* has been found to be fast growing and adaptable to environmental conditions. As such, it has become extensively used in softwood planted forests [33]. An existing wealth of knowledge on host genetics, physiology, production/growth, and health, as well as national and international research trial series (genetics x environment x management) makes Monetary pine a valuable resource for tree microbiome research and comprises an excellent model for gymnosperms more widely.

In this study, we focus on developing a sampling strategy to that allows robust assessment of the bacterial and fungal needle microbiome of *P. radiata*. Given potential role of tree height and canopy influence on microbiome assemblage, a structured approach was followed to quantify how different factors may influence the needle microbiome, and therefore where future studies should focus sampling effort to capture these influences based on their research questions. We focused sampling to a clonally propagated tree of known genetics, age, and management in a commercial forest stand. This uniformity enabled us to minimise potential influences associated with of neighbouring vegetation types, forest composition, etc. By removing such influences, we were able to robustly test for influence of canopy sampling height (bottom, middle and top), cardinal directions (north, east, south, and west), needle age, and surface sterilisation on leaf microbiome richness and assembly.

## Methods

### Sampling strategy

Regard was given to the selection of an appropriately representative *P. radiata* (Monterey pine) specimen and habitat (environmental location) for sampling. The individual selected needed to be in good general health, with no visible needle loss or discolouration, of typical growth form, and being sited within a stand of clonal trees (Supplementary Figs. 1 and 2). This latter criterion ensured that impacts of neighbouring vegetation type would be minimised as neighbouring trees had identical genetic backgrounds. For the site selection, two main criteria were considered. First, the site needed to have an established forest floor layer, thus providing potential source of natural microbiome inoculum for transfer with the canopy (in case this was important). Second, it was essential that no copper or other pesticide had been used for at least 2 years prior to sampling.

Based on this, a six-year-old tree was selected within the commercially operated Kaingaroa Forest, located in the central North Island, New Zealand (176° 22’ 01‘’E; 38° 39’25’’S). The tree was of a known genetic provenance (‘Clone 15’ or, hereafter, C15). Clone C15 has been in commercial use for over a decade and is recognised for relatively rapid growth, desirable wood properties (high moment of elasticity and density), resistance to dothistroma needle blight (*Dothistroma septosporum*), and tolerance to drought [34].

### Site characteristics

The forest plot was scanned using a backpack-mounted mobile laser scanner (Hoevermap; Emesent, Milton, Qld., Australia). Scans followed an approximate path that took in the study tree and the neighbouring trees within the trial area. Using simultaneous localisation and mapping (SLAM), as opposed to global-navigational-satellite system (GNSS), the Hovermap is much more suited to the often GNSS-denied environments in forest stands [35]. Point clouds were generated within the Emesent software package version 1.5 (Emesent, Milton, QLD, Australia) and colourised using the Emesent colourisation kit. The purpose of this work was to provide a future visual reference of the context of the tree and site, not for formal inclusion in data analysis.

### Sampling

**Fig. 1:**
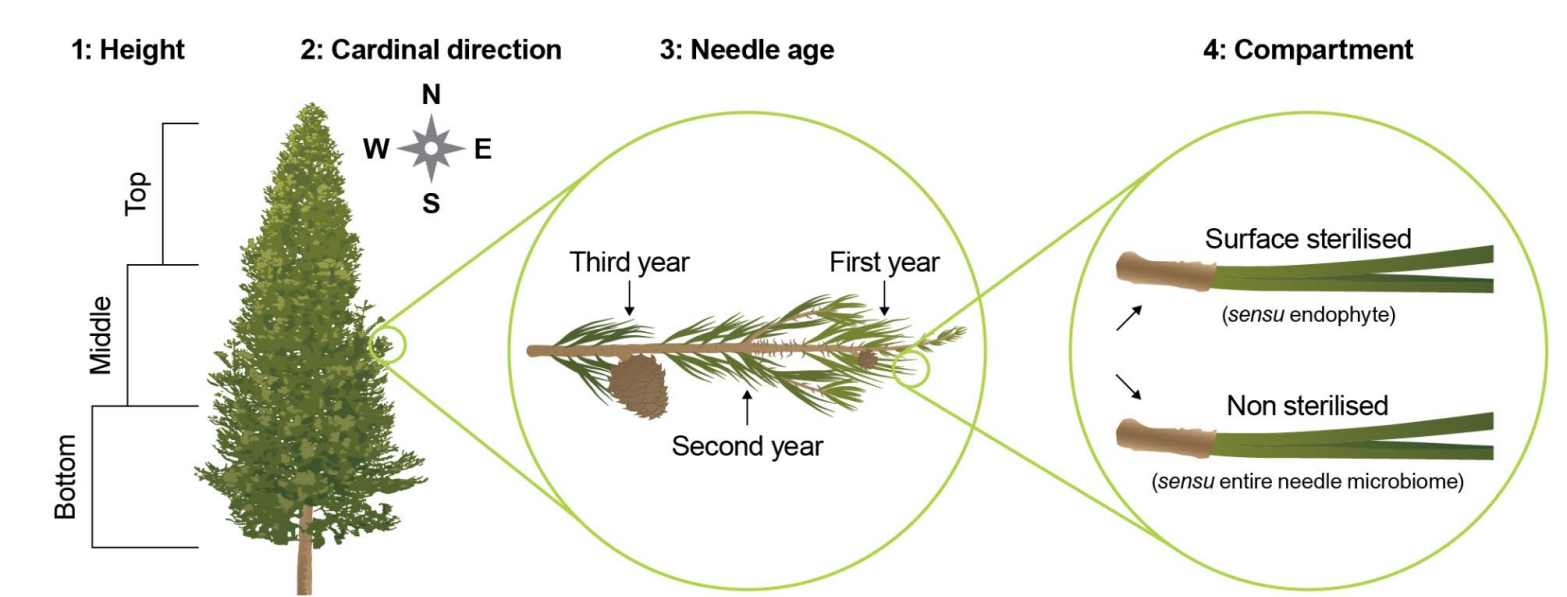
Needle sampling strategy; different tree heights, cardinal directions, needle age, and needle compartment. *note that *Pinus radiata* typically has 3 needles per fascicle. This graphic is for demonstration only

Needle sample collection was undertaken in December (summer) 2019. A structured sampling approach was used to assess the variability of the needle microbiome within the tree canopy. Towards this, two separate fascicles, i.e. 3 needles connected at a sheath (Fig. 1), were randomly collected by sterile technique from within the tree canopy and representing the following treatments: (1) Tree height, comprising the bottom, middle, or top thirds of the tree canopy (approximated from the lowest pruned branch); (2) north, east, south, and west cardinal directions; (3) needles of different ages, based on branch placement, and consisting of year one, year two, and older growth; and, lastly, (4) needles sterilised or non-sterilised before DNA extraction to provide indication of differences in the phylloplane and endophytic ‘compartments’. This sampling strategy, comprising a total of 36 treatment combinations, is illustrated in Fig. 1. For practicability, the needles from one fascicle were used for the sterilised (endophyte) compartment work (i.e., the entire fascicle was surface sterilised; see later), while needles from an adjacent fascicle represented the non-sterilised (phylloplane) data.

Individual fascicle samples were placed into 50 ml tubes, transferred to the lab on ice, and then stored at 4 °C for 2–4 h before processing. Using sterile procedures, each fascicle was divided into its separate needles, each with some sheath remaining. From these, one needle was sliced into small ~ 3 mm diameter fragments for DNA extraction and placed at -20 °C. All work was carried out using aseptic laboratory technique.

We operationally defined the endophyte compartment as the microbiome remaining after washing of needles using a sterilant solution (i.e. physical and chemical processing). Towards this, the second entire fascicle was surface sterilised by placing it in 70% ethanol, washed vigorously for 30 s, then thoroughly washed free of sterilant with rinses of sterile water. A needle from this fascicle was cut into fragments for DNA extraction and frozen (as above).

### Chemical characterisation of needles

For reference purposes, 100 needle fascicles were collected from each of the three canopy levels and processed for nutrient/chemical analysis. These were placed (in bulk) into paper bags, dried at 70 °C to constant mass, and ground to 1 mm in a Wiley mill. Total carbon (C) and total nitrogen (N) were determined using a FPS-21,000 CNS thermal combustion furnace (LECO). Exchangeable Al, B, Ca, Cu, Fe, K, Mg, Mn, Na, P, and Zn were determined by ICP-MS after 1:50 (macro) NH_4_CH_3_COO leaching.

#### Molecular methods and bioinformatics

DNA was isolated from ~ 50 mg finely chopped needle samples using the DNeasy® PowerPlant® Pro Kit (Qiagen) according to the manufacturer’s recommendations. Bacteria- and fungal-specific primer sets were used to amplify the rRNA ITS genes from which the needle microbiome was characterised (i.e., other Eukaryia and Archaea are not considered in this study). Our methodological protocols closely followed those described for the Earth Microbiome Project (EMP) [36].

For the bacterial community, an amplicon library based on the V4-V5 hypervariable regions of the 16S rRNA gene was created using primers 515 F and 806R [37]. The fungal community composition was characterised on ITS gene sequence variation using primers ITS1f [38] and ITS2 [39]. In both cases, primers included Illumina sequencing adaptors and pads, along with a unique Golay 12-mer barcode (on the forward primer for bacteria, and reverse primer for fungal PCR). Barcoding enabled individual samples to be identified following multiplexed sequencing of mixed amplicon pools.

PCRs were conducted over 35 cycles using TaKaRa Ex Taq Hot Start polymerase chemistry (Takara Bio, USA). For 16S rRNA gene amplification, conditions were: 94 °C for 3 min, followed by 35 cycles of 94 °C for 40 s, 50 °C for 60 s, 72 °C for 90 s, and a final extension at 72 °C for 10 min. For amplification of the fungal ITS gene region, dissociation was for 30 s, primer annealing was conducted at 52 °C for 30 s, and extension at 72 °C was for 30 s. All PCRs were conducted on an Agilent AriaMx PCR machine and included eight no-template controls per 96 well plate.

PCR products were purified using a magnetic bead clean-up Kit (Geneaid Biotech Ltd) and pooled, based on standardised DNA concentrations, into a single sample. This was further purified (PureLink™; Life Technologies Ltd) to remove residual magnetic beads.

Sequencing was performed using an Illumina MiSeq system at the Australian Genome Research Facility (AGRF). Bacterial PCR libraries were sequenced using 2 × 250 bp paired end (PE) read chemistry, and fungal libraries using 2 × 300 bp PE chemistry. Paired-end reads were filtered and trimmed using DADA2 [40], implemented with the R environment, using standard filtering parameters (maxN = 0, truncQ = 2, rm.phix = TRUE and maxEE = 2). Samples were dereplicated, paired reads merged, and chimeras removed. Sequences for both 16S rRNA and fungal ITS gene region libraries have been placed in the NCBI sequence read archive (SRA) under the BioProject accession PRJNA672703.

#### ASV filtering and phylogenetic classification

Taxonomy was assigned to the resulting amplicon sequence variants (ASV) from DADA2 using the RDP database release 11.5 [41] or the UNITE database [42] for the bacterial and fungal communities, respectively. Amplicons of plant-based origin (e.g. chloroplasts) dominated the initial bacterial sequencing libraries (85% of sequences) and were removed after taxonomy was assigned; i.e. ASVs where phylum = Cyanobacteria/Chloroplast were discarded. Similarly, ASVs unclassified at kingdom and phylum level, or present as singletons within a sample, were removed from the datasets.

#### Sampling effort, sequencing coverage, and ASV richness (α-diversity)

The estimated richness of fungal and bacterial species (ASVs) in each sample were calculated using the Chao1 index [43]. Interpretation of these were supported via generation of rarefaction curves for each individual sample; i.e. visual inspection to verify asymptotes had been reached (allowing determination if further sequencing of each sample was likely to result in discovery of substantially more taxa). Collector curves were then generated for treatment groups to determine if additional needle sampling would considerably increase the chances of discovery of new taxa. As canopy collection height was later found to be the primary factor associated with both bacterial and fungal needle richness and community structure, data were grouped to this factor, (i.e., all age, direction, and compartment data combined within each height group). These analyses were conducted with the VEGAN package for R [44]. All analysis involving R was conducted using v4.0.0 [45].

Variation in Chao1 indices were tested independently across the treatments using ANOVA, with post-hoc multiple comparisons using Tukey’s test (when 3 or more groups were being compared) or students t-test (2 groups). These tests were conducted in Prism 9.5.1 (GraphPad Software, USA).

#### Bacterial and fungal community composition (β-diversity)

ASV-level data were step-wise aggregated up phylogenetic hierarchy; i.e. ASV to genera, genera to family, and so forth to phylum. Each dataset was standardised and square-root transformed. Individual resemblance matrices were created using the Bray-Curtis (BC) distance method, and similarity among these aggregated datasets then assessed using 2nd stage analysis [46]. This allowed determination of the effect of higher-level aggregation on the behaviour of the data, enabling selection of the highest taxonomic groupings in which biological distances among samples present at ASV level were still preserved. For both bacterial and fungal sequence data, this was Class level.

The influences of height, compartment, cardinal direction, and needle age on microbiome assemblage were assessed using permutation-based multivariate ANOVA (PERMANOVA) [47]. Treatments were ‘fixed’ in the model, permutations (999 x) of the raw data were unrestricted, and sums-of-squares were type III (partial). Where significant main-treatment effects were present (p_perm_<0.05), pair-wise testing within groups was conducted.

Needle samples from the top third of the canopy included many samples with low levels bacterial diversity relative to than those lower in the canopy. Some failed to generate sufficient 16S rRNA amplicons after 35 cycle PCR, or the data returned after sequencing was entirely plant chloroplasts. As these did not pass QC/filtering, there was an uneven distribution of remaining samples among the factors ‘needle age’, ‘cardinal direction’, and ‘surface sterilisation’. As such, interactions among these terms were not able to be robustly tested within the top-canopy. Given this, we conducted an initial main-effects only PERMANOVA to assess the contribution of canopy height, needle age, cardinal direction, and sample sterilisation on needle bacterial β-diversity; these tests are summarised in Table 1. We then conducted a subsequent PERMANOVA after samples from the top-canopy were removed; this second model enabled robust testing of interactions among the different factors on bacterial community assemblages (but only within the middle and lower canopy) needles; these are presented in Table 2.

**Table 1:** Phyllosphere bacterial community composition PERMANOVA results table showing (top panel) all main treatment effects, and (bottom panel) pair-wise testing within canopy heights

Main effects	√CV	ρ
Height of sampling	40.52	0.001
Cardinal direction	5.06	0.102
Needle age	2.37	0.273
Surface sterilisation	-2.40	0.652
Residual	18.24	
**Height of sampling**	**t**	**ρ**
Top v Middle	10.61	0.001
Top v Bottom	11.62	0.001
Middle v Bottom	2.60	0.001

√CV = square root of the component of variation associated for each term

ρ values are the permutation-derived probability statistic. Unique permutations for all tests > 998

**Table 2:** Summary PERMANOVA testing for the effects of main and interaction sampling effects on the composition of bacteria and fungal communities in the *Pinus radiata* phyllosphere

	Bacteria^1^		Fungi	
Test	√CV	ρ	√CV	ρ
Canopy height	**19.20**	**0.001**	**21.42**	**0.001**
Cardinal direction	**14.48**	**0.001**	-2.88	0.610
Needle age	4.94	0.064	-3.33	0.730
Surface sterilisation	4.20	0.075	**6.75**	**0.025**
Height of sampling x cardinal direction	**19.90**	**0.006**	4.42	0.276
Height of sampling x needle age	**18.32**	**0.008**	3.99	0.305
Height of sampling x surface sterilisation	9.07	0.062	-2.21	0.465
Cardinal direction x needle age	5.82	0.222	-6.07	0.823
Cardinal direction x surface sterilisation	5.98	0.147	-5.08	0.746
Needle age x surface sterilisation	3.34	0.234	-3.53	0.569
Residual	77.04		26.09	

^1^ Bacteria are samples from the middle and bottom canopy levels only. Top canopy samples were excluded

√CV = square root of the component of variation associated for each term

ρ values are the permutation-derived probability statistic. Unique permutations for all tests > 998

Visualisation of the effects of sampling on separation among the samples was conducted using non-metric multidimensional scaling (nMDS). All multivariate analyses were conducted in the PRIMER/PERMANOVA + software package using approaches described by Clarke and Warwick (2001) and Anderson et al. (2008) [48, 49].

Heat ‘trees’ for both bacterial and fungal datasets were produced using the R package Metacoder [50]. These were produced to visualise (a) the abundance of different taxa in the top, middle and bottom samples, and (b) the taxa that had significant differences in their relative abundance between the top and bottom, middle and bottom, and top and middle samples. Although previous β-diversity analysis was conducted at class level, the heat-trees were produced using order-level taxa aggregated data. This was conducted to give finer resolution on the taxa that differed among the sample types. Due to the nature of these plots, the class level information is still preserved and apparent. Significant differences between each taxonomic group were tested using Wilcoxon rank-sum test. A false discovery rate (FDR) correction was used to correct for multiple comparisons.

## Results

### Reference material

The chemical status of bulk needles (100), including major and minor plant nutrients, are provided in Supplementary Table S1. The composition of needles from the top of the tree varied across a range of nutrients compared with samples from the middle and bottom canopy. However, as there is a higher frequency of needles are top canopy that are either exposed to full sunlight light and/or dominated by actively growing current-season material, the data maybe more generally indicative of the collection of more metabolically active needle material than a canopy height effect *per se*. As such, the data are provided for reference only and we urge caution in any broader interpretation regarding potential microbiome associations related to these data.

### Site imagery

Imagery of the MLS point cloud, coloured by height, is provided in Supplementary Fig. S1. In addition, a profile view of the trees within the MLS point cloud of the forest plot is provided in Supplementary Fig. S2, and video fly through of the lidar point cloud is provided in Supplementary Video 1.

### ***Pinus radiata*** needle microbiome richness (a-diversity)

Rarefaction curves are presented in Supplementary Fig. S3 (bacteria) and Fig. S4 (fungi). Asymptotes were reached for all samples. For the 16S ASV’s, this required approximately 50,000 sequence reads (i.e., after passing all QC and downstream bioinformatic processing), and for ITS this was ca. 600,000 sequence reads. The conservative observed richness of the fungal needle species, for this plant and collection time, was calculated as 1,677 ASV’s. In comparison, the minimum estimate of bacterial species was 286. The *P. radiata* phyllosphere microbiome was far richer in fungal than bacterial taxa.

**Fig. 2:**
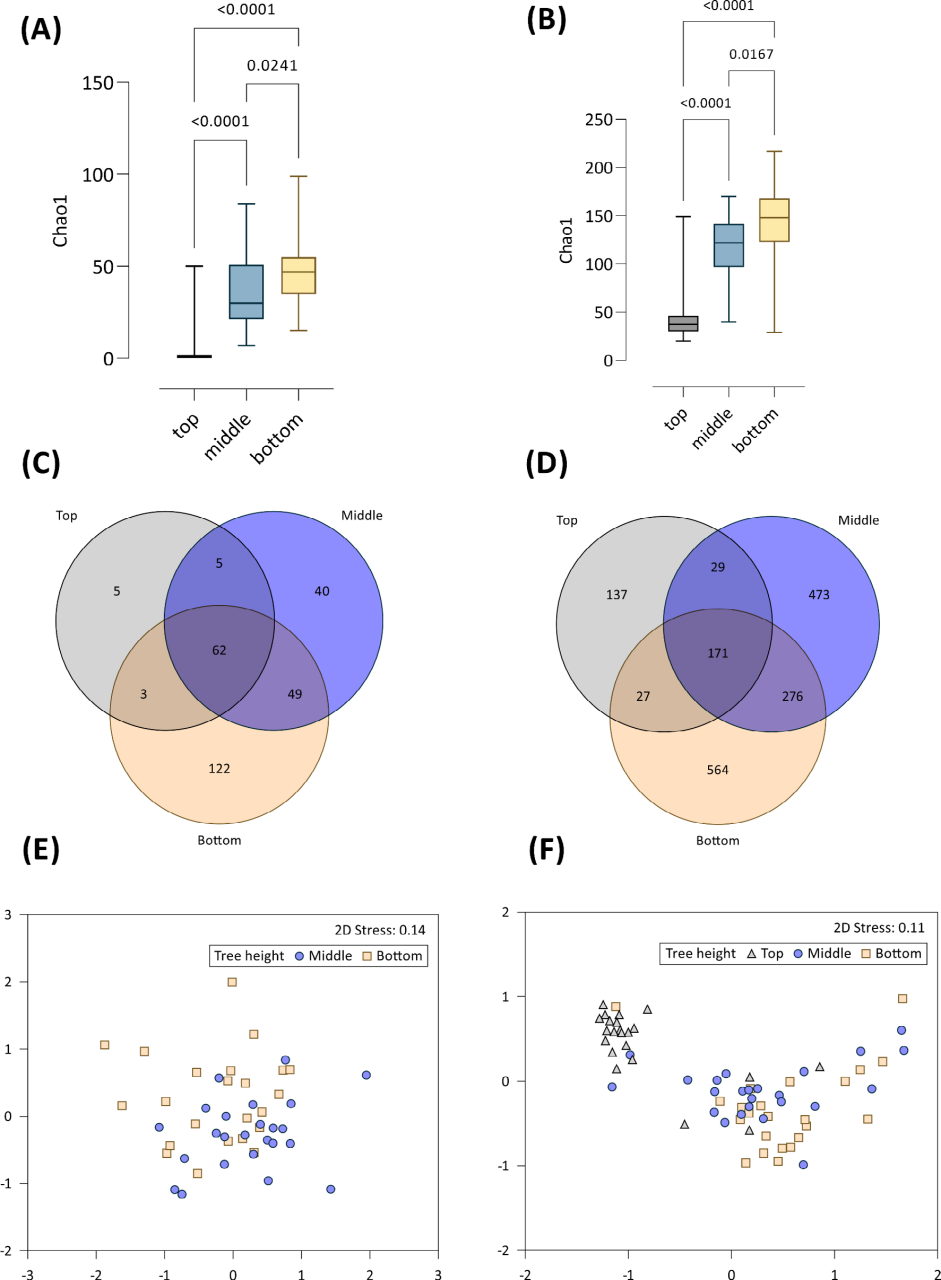
*P. radiata* needle microbiome α and β diversity summary plots at different tree heights. **(A)** bacterial ASV-based Chao1 ‘species’ richness (p = < 0.001), **(B)** fungal Chao1 richness (p = < 0.001), **(C)** bacterial Venn diagram displaying ASVs at three different canopy collection, and **(D)** the fungal Venn diagram also partitioned into canopy height factors. Ordinations (nMDS) using Class-level phylogenetic classification are presented in **(E)** for bacterial communities from the bottom and middle canopy samples only, and **(F)** fungal communities

Variation in Chao1 richness estimates among the canopy treatments were compared with ANOVA and summary results provided in Supplementary Table S2. No association between bacterial or fungal microbiome Chao1 values were present among needles sampled from different cardinal direction, needle ages (branch position), nor if needles were sterilised (all p-values > 0.4). However, height of canopy sampling was important (Fig. 2A and B). For both bacterial and fungal needle microbiomes, species richness is highest in lower parts of the canopy, i.e. closer to the forest floor (Chao1 richness; p < 0.001),

In needles collected from the top third of the canopy, the average fungal species richness was 50.1, increasing to 114.1 in the middle of the tree, and 145.7 species in the lower canopy needles (Fig. 2A and B). While the bacterial community followed the same pattern of increasing richness towards the lower canopy, overall richness per needle was considerably lower than that discovered for fungi (Fig. 2A).

Sharing of taxa among canopy height groups are presented in Venn diagrams in Fig. 2C for bacteria, and Fig. 2D for fungi. Both microbial groups followed similar trends whereby the lowest proportion of unique taxa were present in the top canopy section. Particularly for the bacteria, only five ASVs were exclusively present on needles sampled at the top of the canopy; the majority present in the top canopy were also found in the middle and bottom canopy samples (Fig. 2C). The notable difference between the fungal and bacterial microbiome was the presence of a considerably rich and unique fungal community present in needles from the top part of the canopy; this is also reflected in other data (Fig. 2B). A large component of the fungal community was shared among all compartments (171 taxa; Fig. 2D). Like the bacterial community, however, there was reduced extent of sharing in community present in only the top and middle, or top and bottom samples.

#### Structural composition of the needle microbiome (β-diversity)

Canopy sampling height was the primary (but not only) determinant associated with variation in the composition of the microbiome on *P. radiata* needles. The summary PERMANOVA effects are in Table 1 (all bacterial samples), and Table 2 (bacterial samples from middle and bottom canopy sections only; see explanation in the methods section). The associated ordination plots (nMDS) showing similarity among samples are given in Fig. 2E and F.

For the bacterial community, main-effects testing demonstrated the overarching influence of canopy height (√CV 40.52, p = 0.001; Table 1). Indeed, the vast majority of the variation in the model was accounted for by canopy height (total absolute √CV in the model, including residuals, was 68.59). Subsequent pair-wise testing found each of the collection heights to be statistically different (p < 0.05), however the strongest differences were evident when bacterial communities in needles collected from the top of the canopy were compared with those from the middle and lower sampling regions (Table 1).

Subsequent analysis of the bacterial community was conducted with samples from the top canopy excluded. This allowed for robust testing of interaction effects, but only within the middle and bottom canopy samples. Interestingly, the exclusion of the top-canopy samples enabled discovery of a latent main-effect of cardinal direction (√CV 14.48, p = 0.001; Table 2), and this differed between middle and bottom canopy samples (interaction p = 0.006). Furthermore, an influence of needle age on bacterial community composition was present, but only in interaction with the two canopy heights (p = 0.008; Table 2).

Although these interactions were present, the first-order effect of canopy sampling height on bacterial phyllosphere microbiome remained. In the ‘all data’ nMDS ordination plot (Supplementary Fig. S5), the middle and bottom canopy samples effectively collapsed to a single point as the upper canopy samples were so strongly dissimilar in community composition. The dissimilarity was likely driven by low relative species richness present in the top canopy needles (Fig. 2A). While most taxa present in the upper canopy were also represented in the middle and lower canopy samples, the reverse was not true (Fig. 2C). A secondary nMDS plot was generated, with the highly dissimilar top-samples removed, to allow visualisation of distance among bacterial microbiome from the middle and bottom needle samples; this reduced nMDS is presented in Fig. 2E.

Variation in the fungal phyllosphere community was also primarily associated with height at which needles were sampled (√CV 21.42, p = 0.001; Table 2). The variance associated with this single factor was far greater than all other model terms evaluated, including interactions (see √CV values, Table 2). Secondary testing (not shown) found this influence of needle height was differences between the top and other canopy locations (p = 0.001 for both), but the difference between the middle and bottom needles was low (p = 0.083). These effects are evident on the nMDS ordination in Fig. 2F. A weak, secondary influence of needle sterilisation on fungal community composition was also present (√CV 6.75, p = 0.025; Table 2). No interaction or other effects were present (Table 2).

#### Taxonomic composition of microbial communities at different needle tree heights

Bacterial (16S rRNA gene) taxonomic classification at class level showed numerous small changes associated needle height in the canopy (Supplementary Fig. S6). Overall, however, the most dominant class present on needles was Alpha-proteobacteria, followed by Acidobacteria Gp1, and then other groups.

**Fig. 3:**
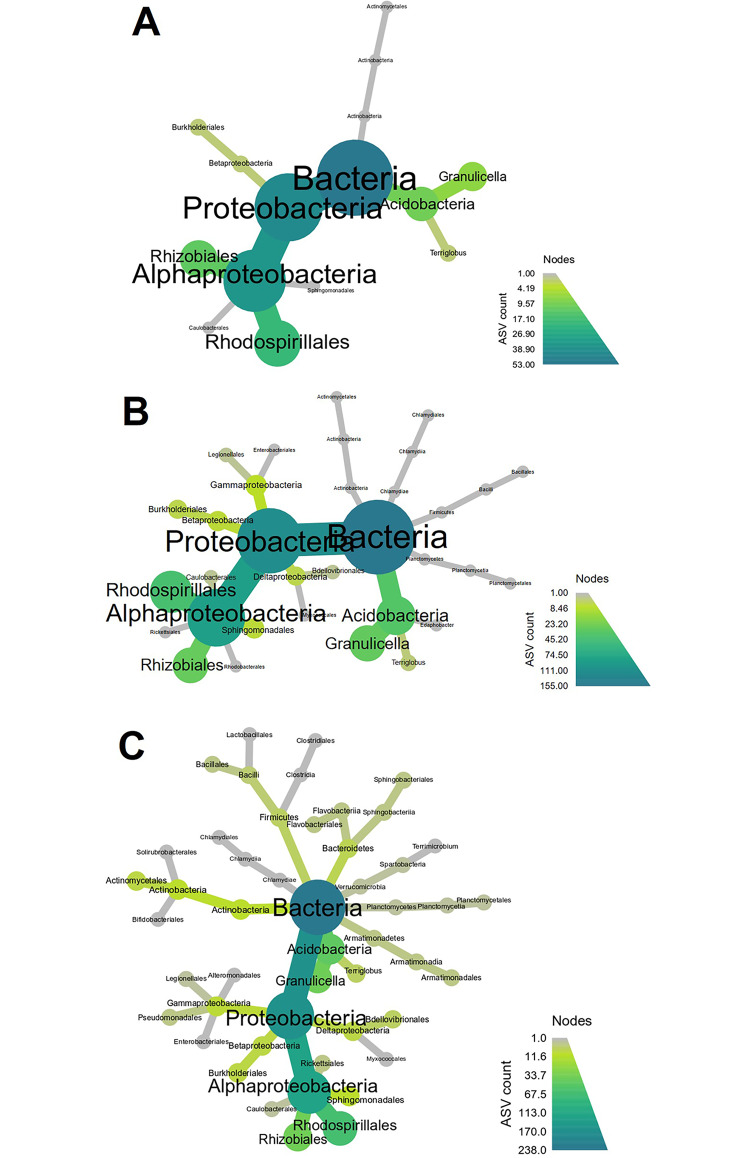
Heat trees based on bacterial taxa counts at order level from needles collected from the **(A)** top, **(B)** middle, and **(C)** bottom portions of the *P. radiata* canopy. The size and colour of the nodes and edges are correlated with the abundance of bacterial taxa in the community

**Fig. 4:**
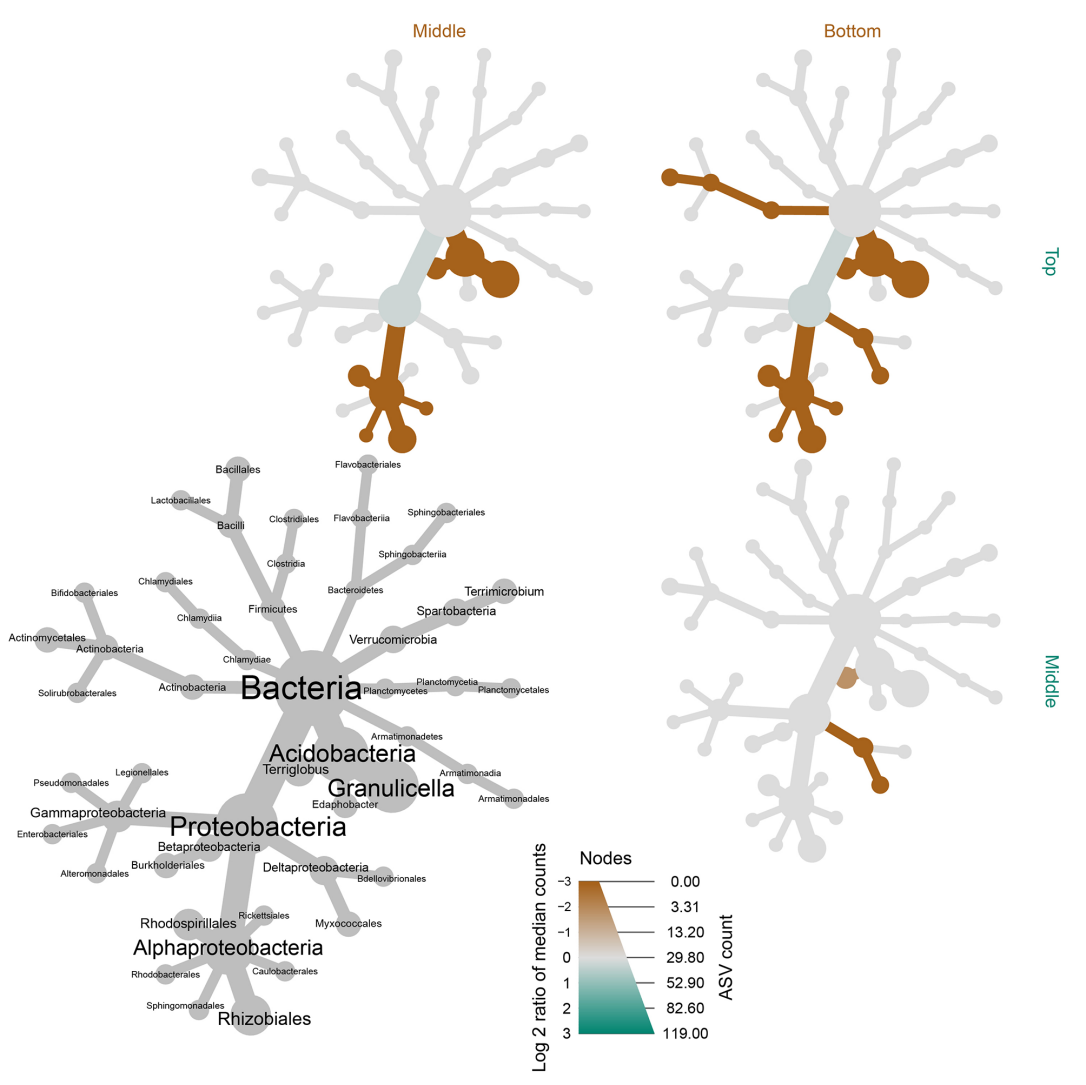
Heat map of pairwise comparisons of the *P. radiata* needle bacterial microbiome among different canopy heights from a *P. radiata* tree. Only significant differences are coloured, determined using a Wilcox rank-sum test followed by a Benjamini-Hochber false discovery rate (FDR) correction. Taxa coloured green are enriched in the part of the tree shown in the row (i.e., none in this case) and those coloured brown are enriched in the part of the tree show in the column

Heatmap trees were used to determine abundances of groups at order level (Fig. 3). Similar to previous findings, key changes in the phyllosphere community were associated with differences between the top and middle, and top and bottom of the canopy (Fig. 4). The results show a conserved group of bacteria was present in the phyllosphere of needles throughout the tree, with key differences in the presence of a few groups in the middle and bottom portions of the tree. A pairwise comparison shows the bottom and middle of the tree were enriched in bacteria relating to Rhizobiales, Terriglobus, and Granulicella, with needles from the lower canopy also enriched in Myxococcales and Actinomycetales (Fig. 4).

ASV analysis identified the most abundant ASV belonging to the order Rhizobiales (ASV 5) with the second most abundant matching to the genus *Sphingomonas* (ASV 7). ASV 5 was the most abundant ASV within the bottom of the tree representing 18% of the total bottom-canopy sequences. In the middle and top canopy samples, ASV 7 was the most abundant with 10.3% (middle) and 27.6% (top) abundances respectively. A full list of taxa identified for each ASV can be found in Supplementary data.

**Fig. 5:**
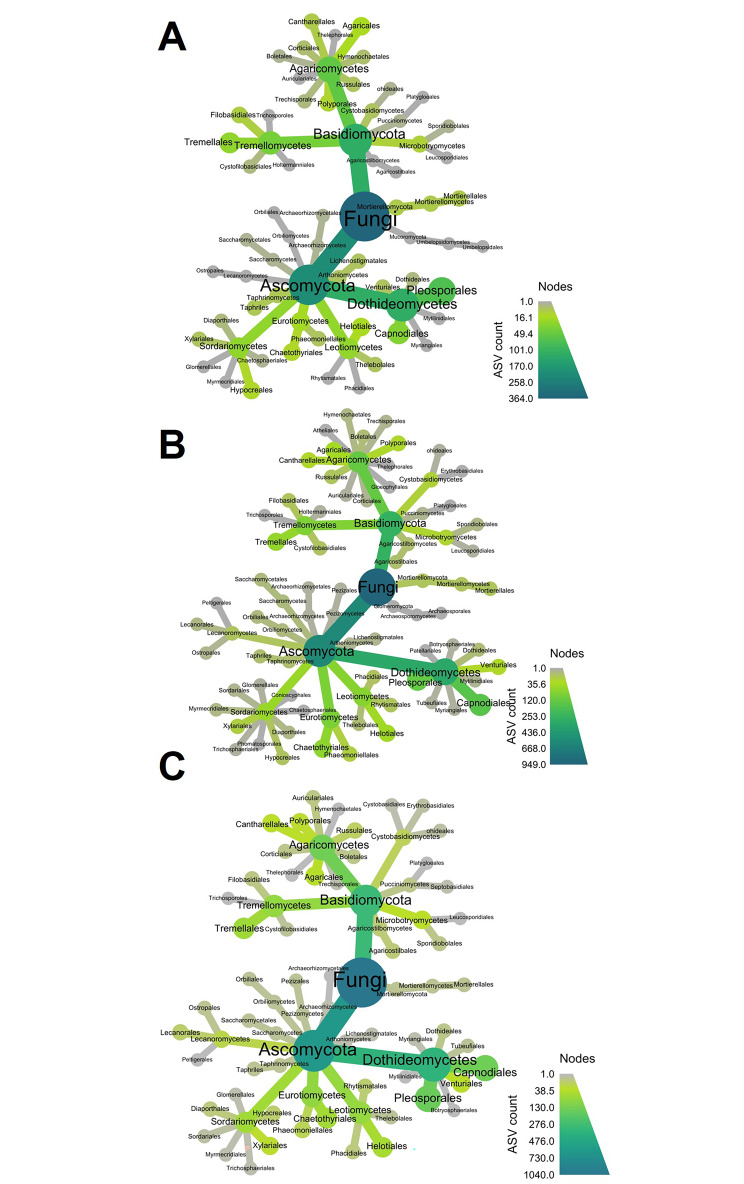
Heat trees based on ASV counts of fungal taxa at order level from needles collected from the **(A)** top, **(B)** middle, and **(C)** and bottom portions of the *Pinus radiata* canopy. The size and colour of the nodes and edges are correlated with the abundance of fungal ASVs in the community

Fungal (ITS gene) taxonomic classification at class level showed large changes in the community across canopy height (Supplementary Fig. S7). There were clear differences in the fungi present as well as their abundances for each collection height (Fig. 5). Needles from the top of the tree hosted a large proportion of Arthoniomycetes and Dothideomycetes. The relative abundances of Arthoniomycetes reduced with distance down the tree, with lowest abundance in the bottom canopy component.

**Fig. 6:**
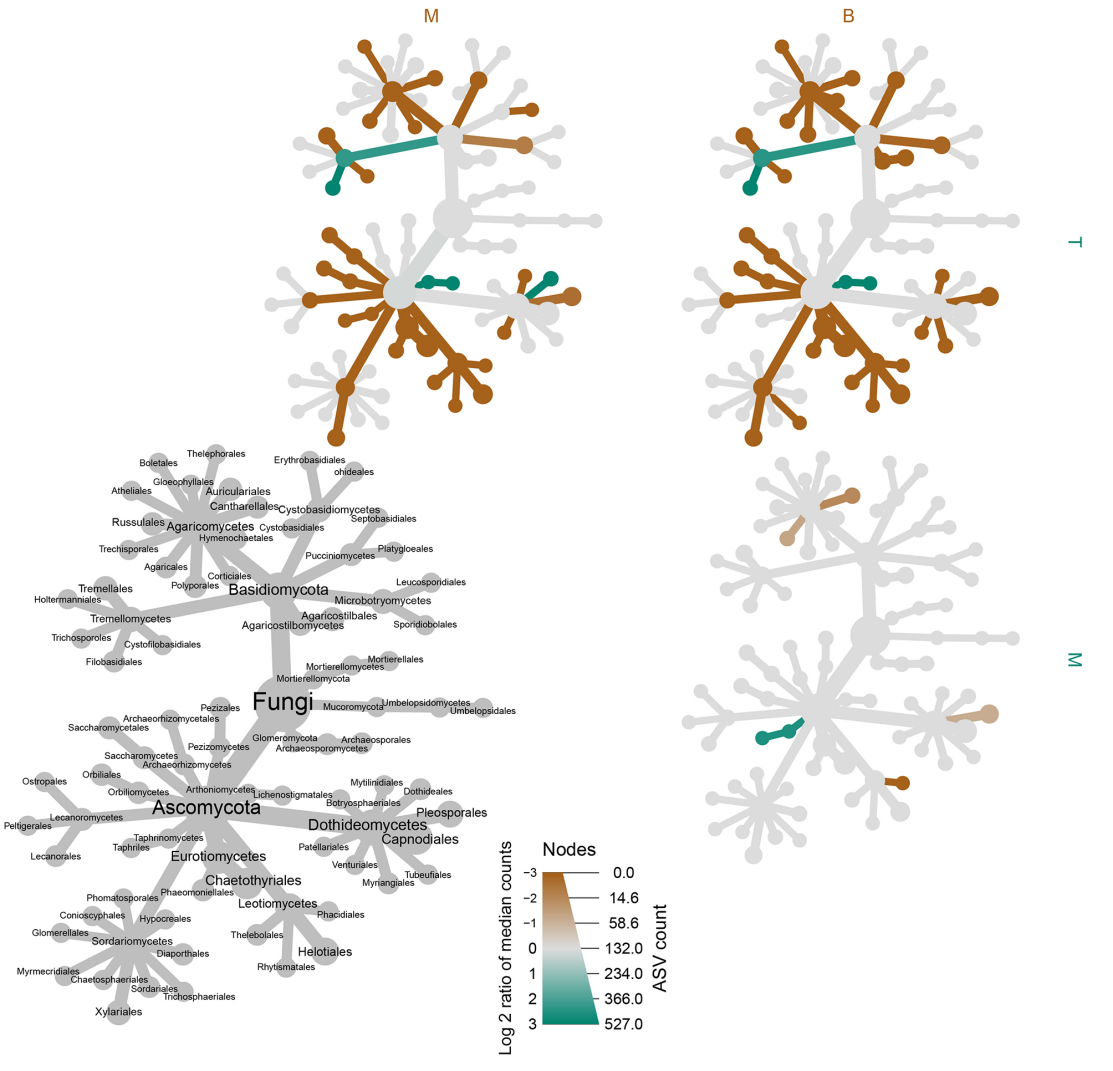
Heat map to show pairwise comparisons of the needle fungal communities in different canopy height sections of a *P. radiata* tree. Only significant differences are coloured, determined using a Wilcox rank-sum test followed by a Benjamini-Hochber FDR correction for multiple comparisons. Taxa coloured green are enriched in the part of the tree shown in the row and those coloured brown are enriched in the part of the tree show in the column

Pairwise comparison of the different tree heights shows that the middle and bottom of the tree shared a similar fungal community, but both differed significantly to the top of the tree. The bottom and middle were both enriched in Ascomycota. These included Xylariales, Helotiales, Boletales and Tremallales (Fig. 6). The top of the tree was enriched for Tremellomycetes, Filobasidiales, Lichenostigmatales, and Dothideales (Fig. 6).

The most abundant fungi belonged to the genus *Phaeococcomyces* (ASV 1; 14.4% total dataset) with the second most abundant matching to the species *Phaeotheca fissurella* (ASV 2; 10.38% total dataset). ASV 11 was the most abundant ASV within the bottom of the tree representing 9.2% of the total bottom canopy sequences. ASV 3 represented the most abundant ASV for the middle of the tree 12.8% and ASV 1 represented the most abundant ASV for the top of the tree 35.0%. A full list of taxa identified for each ASV can be found in the Supplementary data.

## Discussion

This study focused on *Pinus radiata* as a model system to develop standardized and representative sampling approaches for evaluating the phyllosphere microbiome of coniferous trees. This research is significant due to the extensive global coverage of forests, the potential risk posed to them by various biotic and abiotic stressors, and the need to sustain the delivery of services that these ecosystems provide. The boreal forest biome, for instance, is particularly sensitive to climate change [29]. Moreover, sustainable production of planted forests, including those based on Gymnosperms such as *Pinus* spp., is vital to meet the demands of a growing population and the global shift towards bio-based economies [33]. These forests provide wood, fibre, and fuel, thereby reducing the pressure on old-growth natural forests and leaving more space for nature. Additionally, the use of microbiomes to enhance tree health and fitness has the potential to generate new opportunities for forests and ecosystems similar to the human microbiome project for people [51–53].

### Differences in microbial diversity and composition between needles from different tree heights

The canopy height at which needles were collected exerted the greatest influence on the microbial community in the *P. radiata* phyllosphere. This was primarily expressed through a reduction in microbiome diversity with increased canopy height. The sharp distinction between microbial communities (both total richness and species present) associated with the top and lower canopy sampling positions was somewhat surprising given that there was only a 2 m gap between collection heights. However, consideration of the differences in the abiotic environmental conditions with increasing tree height provides some explanation for this observation. This finding indirectly supported the hypothesis that microbiomes from the ground (forest floor, soil) would be a key source of recruitment into the *P. radiata* tree canopy. The forest litter layer and soil are natural reservoirs of a wide range of microbes, and it is reasonable to assume greater transfer of microbial species to the sections of the tree closer to the ground [54]. Such transfer of microbiomes may naturally occur through air flow in forests. Lower branches, for example, can insulate the forest floor and create warm pockets of air that circulate between the ground and the lower branches. This can provide a mechanism to transport bacterial and fungal spores onto the foliage [55, 56]. The importance of soil as a reservoir for phyllosphere membership is supported in other plant systems (e.g., [16], [57]). While we don’t yet have direct evidence for this for *P. radiata*, long-term assessments of microbiome connectivity within forest comportments – soils, needles, aerobiomes and so on – are underway.

Climate can be a key factor in determining the structure and function of microbial communities [58–60]. In a stand of trees, the top of the canopy is exposed to extremes in light intensity, temperature, moisture, and airflow. In the lower canopy, the greater needle mass creates an insulated and more stable environment. This may enable development and persistence of a more extensive range of niches for microbes to inhabit, supporting the observed greater diversity. For instance, Gervers et al., (2022) used LiDAR to assess the degree of canopy closure (density) in *Pseudotsuga menziesii* (Douglas Fir) in relation to fungal microbiome assembly [61]. The authors found that increased canopy closure accounted for more variation than height per se, and that effects of needle age were expressed within the closed-canopy microenvironments. Given the relationship between crown closure and sampling height, it is likely the two factors are entangled: a portion of the canopy height effect observed in our study is likely related to canopy density/closure. There may be some evidence for this in the data; at lower canopy levels, an influence of needle age was evident on bacterial community composition. However, this cannot be definitively partitioned from other influences such as distance to canopy edge and environmental exposure, nor transmission of microbiome from forest-floor and soil to the lower and denser canopy areas. In short, tree canopies are a structurally complex and dynamic habitats, and this complexity needs to be embraced in further microbiome studies. Tools such as LiDAR used by Gervers et al. [61] and now in this study will greatly assist in capturing such complexity.

### Key microbial taxa in the phyllosphere

Alpha-proteobacteria were the dominant bacterial class in the *P. radiata* phyllosphere, and the most abundant order within these were Rhizobiales. Rhizobiales have been found to be associated most often with soils and roots. They are known atmospheric nitrogen fixers when in symbiosis with leguminous plants, but they have also been found associated with many non-leguminous plants such as *Pinus* species [62, 63]. It has been previously shown that phyllosphere bacterial communities are generally dominated by Proteobacteria, and particularly Alpha-proteobacteria [2], however the role or association of Rhizobiales and the host plant has yet to be determined. Given the extent of literature on Rhizobia-legume interactions and N_2_-fixation, it is natural we might initially focus on the N-fixing ability of these bacteria, perhaps in a free-living or non-nodule forming relationship with the plant. However, this distracts investigation into novel and alternative associations Rhizobiales may have with trees and in forest ecosystems more widely.

Bacteroidetes have been demonstrated to colonise the phyllosphere of tree species. A study by Redford et al. [64] found that representatives of Bacteroidetes accounted for 22.5% of the total sequences from samples across 56 tree species. While the research did include a number of *Pinus* species, *P. radiata* was not included. Within our study, the abundance of Bacteroidetes was negligible, representing only 0.38% of total phyllosphere sequences (all within an individual sample). *Bacteroidetes* have been reported to be more abundant on conifers than angiosperms, representing up to 70% of sequences from leaves [65]. It is unclear why Bacteroidetes are at such low abundance in the *P. radiata* phyllosphere. It is possible our selected trees or sampling environments were unique/anomalous, however specific consideration was given to sampling typical specimens in standard environments. Only further assessments can determine this.

The most abundant class of fungal species within the phyllosphere belonged to the Dothideomycetes. This fungal class was abundant on needles irrespective of the canopy height sampled. Dothideomyceta contain diverse species from a range of environments and are loosely defined as plant associates and often encountered as saprophytes of dead leaves and wood material [66].

Changes in canopy height was related to significant shifts in abundance of classes of other fungal taxa, i.e., different fungal ASVs dominated at different heights. This finding concurred with another study identifying the fungal portion of the microbiome from *Pinus nigra* [67]. The authors found that fungal species richness was not affected by site, or the individual tree sampled but varied with tree height and among forest patches. Our study found similar trends with both fungal and bacterial communities.

Taudière et al. (2018) [67] also found five out of the ten key foliar endophytes identified in a review by Sieber (2007) [60] as key pine symbionts. This review identified Dothideales and Helotiales as dominant fungal orders in a range of studies, however most were identified through the use of culture-based techniques. These fungal taxa were identified as endophytes where our study looked at the entire needle phyllosphere. The developing area identifying the key microbiomes from *Pinaceae* needles for both fungal and bacterial communities demonstrate an important literature gap for better understanding of the mutualistic benefits of microbes and their foliar counterparts.

Previous studies have provided some insights into the scale of variation in the needle microbiome, but these studies have generally only focused on one part of the microbiome rather than understanding the whole phyllosphere. Yet understanding the role, diversity, transmission, and interactions of the microbiome colonising the phyllosphere of *P. radiata* is important. Not only can this fundamentally impact plant health (disease to physiology), but extends well beyond the plant itself to ecosystem-level processes such as the cycling of carbon and nitrogen. In a multi-host study, Carrell et al. [54] studied the needle endophyte microbiota of *Pinus flexilis* and *Picea engelmannii*. They found a consistent needle endophyte microbiota with the majority of taxa belonging to two bacterial phyla - Acidobacteria and Proteobacteria - with the key phylotype *Gluconacetobacteria diazotrophicus* and other nitrogen-fixing bacterial endophytes dominating. A few fungal specific studies have focused on the diversity of foliar endophyte ascomycetes in pine forests. Taudière et al. [67] found that tree age and forest patches created the most difference across sites and tree cohorts. One study by Rúa et al. (2016) [68] investigated the potential role ectomycorrhizal (ECM) fungi play in structuring the foliar bacterial endophyte communities of *P. radiata*. They suggest that the ECM fungi may be an important factor for explaining the variation in bacterial endophyte communities, but the effect was influenced by population and environmental characteristics. This highlights the importance of studying interactions, including between biotic and abiotic factors in structuring plant microbiome communities more generally, and the need for appropriate model systems to test these.

### Shared microbiome membership

Shade and Handelsman (2012) describe the core microbiome as ‘members common to two or more microbial assemblages associated within a habitat’ [69]. Based on this definition, we observed many fungal and bacteria to be consistently present within the tree canopy (i.e. shared among samples), giving supporting to the concept of a core phyllosphere microbiome of *P. radiata*.

Within the fungal community, 10.2% of ASVs were shared across the whole tree. For the bacterial community, this was 21.6% of ASVs. Being able to identify the core microbiome is a key step towards prediction of the primary functions the phyllosphere microbiome may confer to the host. These maybe inferred, for example, through DNA analysis of the genomes of these taxa. However, this will necessitate isolation and cultivation of these taxa and genome sequencing. In these systems, metagenomic methods are unlikely to be effective. For example, the *Pinus radiata* genome is approximately 25 billion base pairs in size [70] (or ~ 8 x the human genome); the detection let alone assembly of microbial genomes that are 1000’s of times smaller and far less abundant in DNA concentration than that of the host, will be challenging (and expensive). Regardless, the benefit of conducting culture-based isolation and genome sequencing is the capacity to gain direct information from the culture itself, from fundamental physiological information (e.g. pH range, optimal temperature), through to being able to explore interactions with the host and environment which only experimental testing can determine.

The separation of the phyllosphere microbiome into core and variable components also allows for partitioning the role of factors spanning host genetics, environmental conditions, through to forest management, priority effects, and stochastic processes on the phyllosphere microbiome. For example, we would expect that factors directly impacting host physiology would be expressed most strongly on the core microbiome [71]; these may have resulted due to co-evolutionary processes and therefore changes in the core microbiome will be most attuned to changes in the host genetic state. Conversely, environmental or management factors being expressed on the tree, could result in dysbiosis to the core microbiome increasing the risk of disease expression (e.g. Arnault et al. [72]). A stable core microbiome provides insights into the average community and therefore a possible standard for predicting community responses to various disturbances. Our findings demonstrate a shared membership presence for both prokaryotic and fungal communities, spanning the full tree height with more differences associated with the middle and bottom of the tree compared with the top.

### Methodological and experimental considerations

The primary goal of this work was to test and validate a methodological approach for the robust sampling of the phyllosphere microbiome in coniferous trees. Towards this, our study focussed on the within-canopy microbiome of a single tree. We attest that focus on a single tree can be as meaningful to microbiome and microbial ecology studies as tree mensuration/growth studies are when conducted within a single forest. For the latter, a single forest would be typically divided into experimental units enabling a level of replication. For example, these could be geographically defined areas such as catchments, or other macroecological zones or gradients through to anthropogenically defined and delimited stands of trees. For the microbiome within the tree canopy (i.e., *sensu* a ‘forest’ equivalent for microbes) we have conducted similar delimitation, *a priori* delimiting the canopy into heights, aspects, and so forth, and then replicated sampling of needles within these.

From a microbial perspective, the canopy of a tree and its associated phylloplane area is an incredibly vast ‘forest’. For microorganisms with body sizes in the µm scale range, a single needle that spans cm’s of length is 10^4^ x equivalent body size. This is a considerable relative distance and one that, in some regards, becomes irrelevant for key parts the organisms’ ecology. For example, microorganisms residing on opposite sides of the needle surface or colonies present at either ends of the needle, are effectively isolated from each other by the relative distance between them. This precludes direct or physical interactions, competition for resources, etc. Key aspects of their ecology and particularly interaction with the host and environment need to be assessed at much finer spatial scale. In our study, when the sampling unit comprises entire needles, we are inevitably overlooking much of the relevant ecological context that is associated with the distribution and functioning of phyllosphere microbial community [73]. I.e. it is simply too big to capture the scale of influences that impact many aspects of the ecology of microorganisms phyllosphere microbial communities. An alternative perspective could be, why wasn’t this study all conducted on a single needle, as the tree is simply too large and diverse in habitat types as to lose meaning? The answer, of course, that we need to look across all these different scales to understand the roles of different drivers phyllosphere ecology, from dispersal processes and limitation that might occur among leaves and between canopies, through to fine-scale microbial interactions and signalling on the leaf surface. Thus, what we consider to be our experimental unit must adapt and be appropriate for the research question.

Targeting bacterial microbiome detection using PCR amplification from plant tissues has been shown to be problematic. Often primers amplify ‘contamination from plant genes’ such as chloroplast DNA. These are, of course, examples of ancient bacterial endosymbiosis [74]. While the detection of these can be frustrating in some situations, it can alternatively be viewed as wonderous that the 16S rRNA sequencing tools available can peer back into evolutionary history and the role of microbiome associations as fundamental to the eukaryotic life we see today. Further exploration to understand how microbiome associations influence the tissues, physiology, and ecology of plants is central to many of the studies being conducted presently. Yet, in a practical sense, an outcome for researchers and bioinformaticians can be an overwhelming prevalence of ‘plant origin’ amplicons in 16S rRNA libraries. We conducted background experiments with and without plant blockers [e.g., [75]] on some of the *P. radiata* foliar DNA samples. Regardless, the MiSeq libraries remained populated with chloroplast sequences. We note other studies have explored use of alternative primers with bias against chloroplasts [76]. However, we chose to retain use of the widely used 515F and 806R primers [37] and tested the extent of sequencing depth required to provide coverage of prokaryotic taxa once ‘plant origin’ reads (~ 85% of sequences) were removed. The outcomes of these are provided in the supplementary information (Fig. S1). For this study, all sequencing reached asymptote indicating coverage of expected ASV variants.

An additional part of the methodology identified a sterilising method for identifying endophytes vs. epiphytes. The method chosen was of mixed benefit at removing the DNA from the external surfaces (an ethanol wash vs. a bleach DNA removal). Its application did not result in significant differences for the bacterial libraries but was more successful at removal of fungal DNA from the needle surface resulting in significant difference between sterilised and non-sterilised samples. There are numerous studies in the literature that describe a variety of methods and results to clearly identify endophytes from tree tissues. This needs further structured exploration to determine the efficacy of different methods within a more targeted study.

### Enabling robust future sampling

The sampling strategy we conducted was towards two key outcomes. Firstly, from a microbial perspective, we were able to explore factors associated with the distribution of the conifer phyllosphere microbiome at tree-level. That is, drivers of the patterns of occurrence and structure of the microbial communities when a canopy ecosystem is divided into different niches. First-order drivers of canopy factors on phyllosphere microbiome communities were evident: primarily the height of canopy sampling and, secondary to this, influence of cardinal collection or needle sterilisation. Secondly, our sampling allowed us to assess the extent of variation in microbiomes among sample types, how species rich samples from different within-canopy niches are, and the extent of DNA sequencing needed to reach coverage (asymptote) of these. The combined knowledge of where and how many in the canopy to sample is important. It enables robust capacity to conduct experiments focussed *within* the canopy itself, for example evaluating the role of UV exposure on filtering microbiomes in the top v lower canopy. Our work also enables robust planning of experiments operating within the canopy-level experimental unit. Examples could be assessing tree-to-tree variation in phyllosphere microbiomes, quantifying the role of host genetics in microbiome association, testing the role of silviculture and forest management and so forth. In these instances, understanding the requirements for collection of a representative ‘canopy-level’ microbiome sample can be essential. If collection of needle samples is not standardised from a canopy location, the influence of subtle factors such as tree genetics may not be detectable within the variation caused by random or non-structured canopy location collection.

Finally, and as discussed previously, a missing gap from our work is assessment of phyllosphere microbiome variation at the individual needle scale. From a microbial perspective, a single needle presents and large and diverse ecosystem in its own regards. Many key factors influencing the overall assembly and functioning of the phyllosphere microbiome of trees may be driven by processes occurring on discrete sections of the needle. By pooling entire needles for DNA extraction, we lose the opportunity to discover where and subsequently how key ecological filtering occurs, nor where microbial interactions and priority effects are expressed. For such ecological processes, let alone microbial interactions with the tree tissue and concomitant phenotypic changes or fitness outcomes, the appropriate ecological level is the needle itself, or a discrete niche or tissue within or upon a needle. In summary, we must work at valid scales appropriate to the ecological questions being tested.

### Future research

The methods described here provide an understanding of the microbiome differences across a whole tree canopy phyllosphere and where the largest extent of variation can be expected when sampling. The approach presented can facilitate design of robust experiments that enable the understanding of phyllosphere microbiomes, their biodiversity, ecology, and interaction with the host plant.

Towards the development of *Pinus radiata* as a model conifer tree species for microbiome research, focus on ecology at both finer (within needle) and broader (among trees and forests) scales are important. By looking across all scales, we aim to build an understanding of the environmental, genetic, stochastic and other factors shaping phyllosphere community assembly and function, and how these impact the fitness of the *Pinus radiata* holobiont in a changing climate.

## Electronic supplementary material

Below is the link to the electronic supplementary material.

Supplementary Material 1

Supplementary Material 2

Supplementary Material 3

## Data Availability

The data that support the study are in the article and supplementary materials. Raw sequences are available at the National Centre for Biotechnology Information Sequence Read Archive (SRA) under the BioProject accession PRJNA672703.
