# Supplementary material for: What matters most? Assessment of within-canopy factors influencing the needle microbiome of the model conifer, Pinus radiata
Source: Environ Microbiome. 2023 May 30;18:45. doi: 10.1186/s40793-023-00507-8 (PMC10230745; doi:10.1186/s40793-023-00507-8)
Supplement: Supplementary file 2 — Supplementary Material 2 [file 40793_2023_507_MOESM2_ESM.docx]

Supplementary Figures


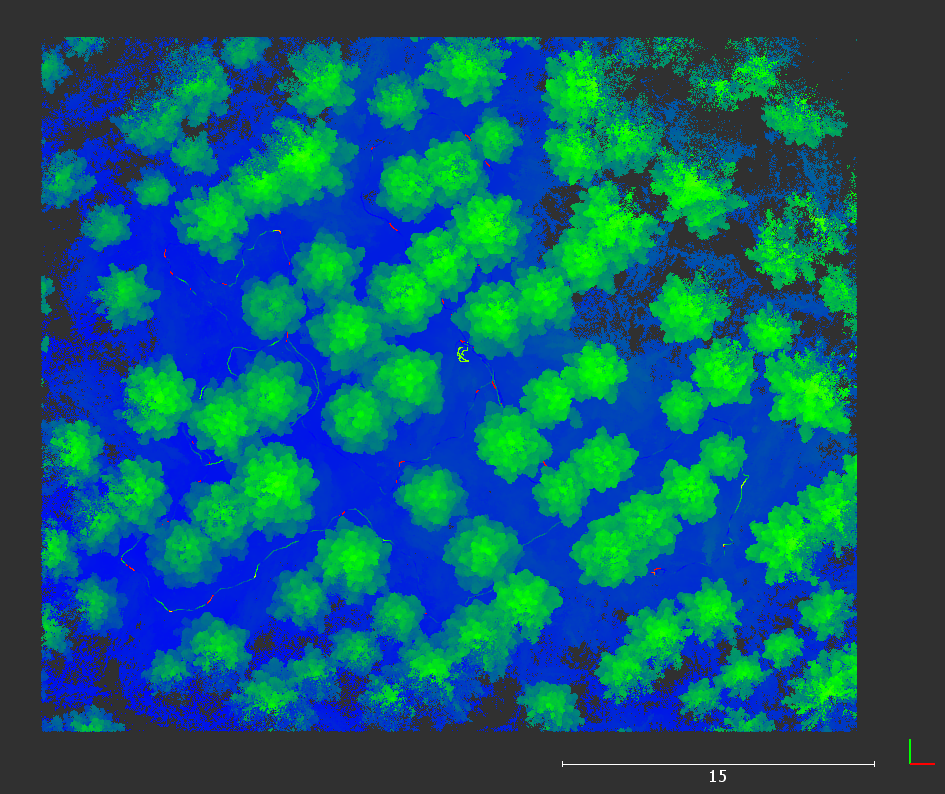


**Supplementary Fig. 1. Nadir i**mage of the MLS point cloud, **showing the canopy cover within the forest plot within the stand. Point cloud is coloured by height, with high points, in this case tree canopies, represented in green. Scale bar in metres.**


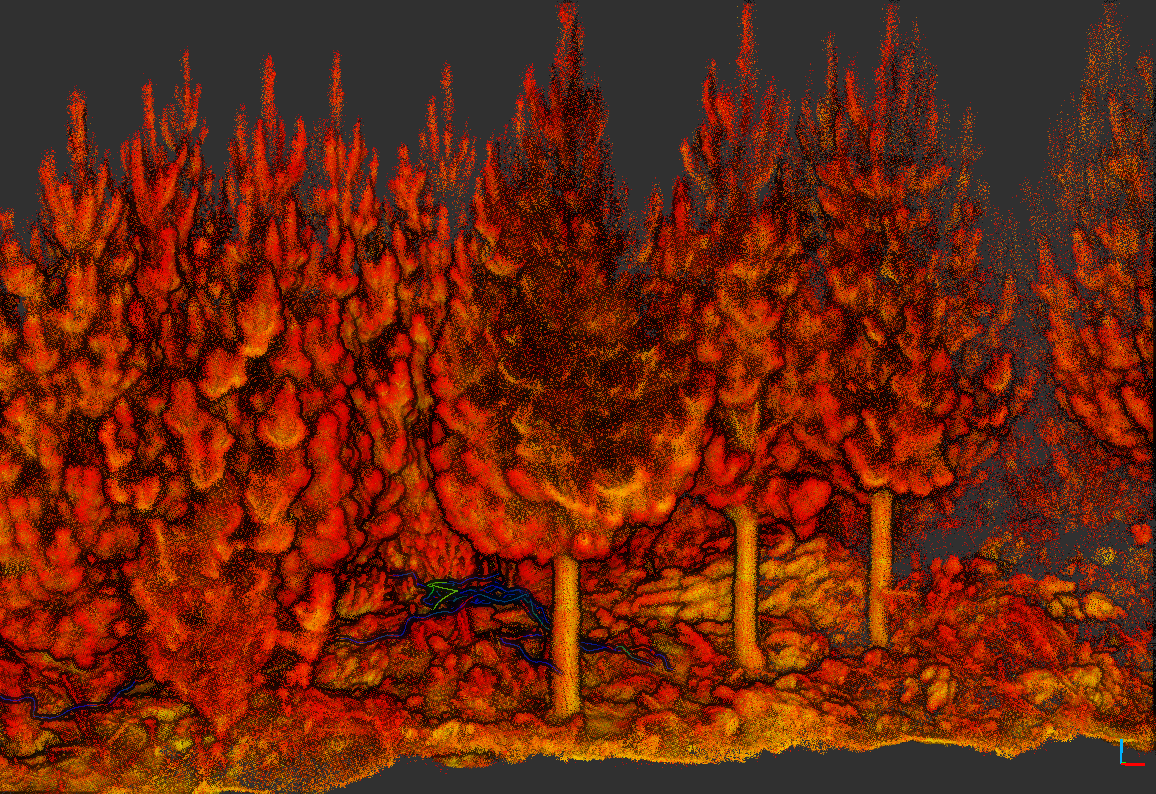


**Supplementary Fig. S2. Profile view of the MLS point cloud showing the forest plot within the stand. The *Pinus radiata* specimen sampled is in centre image.**

| 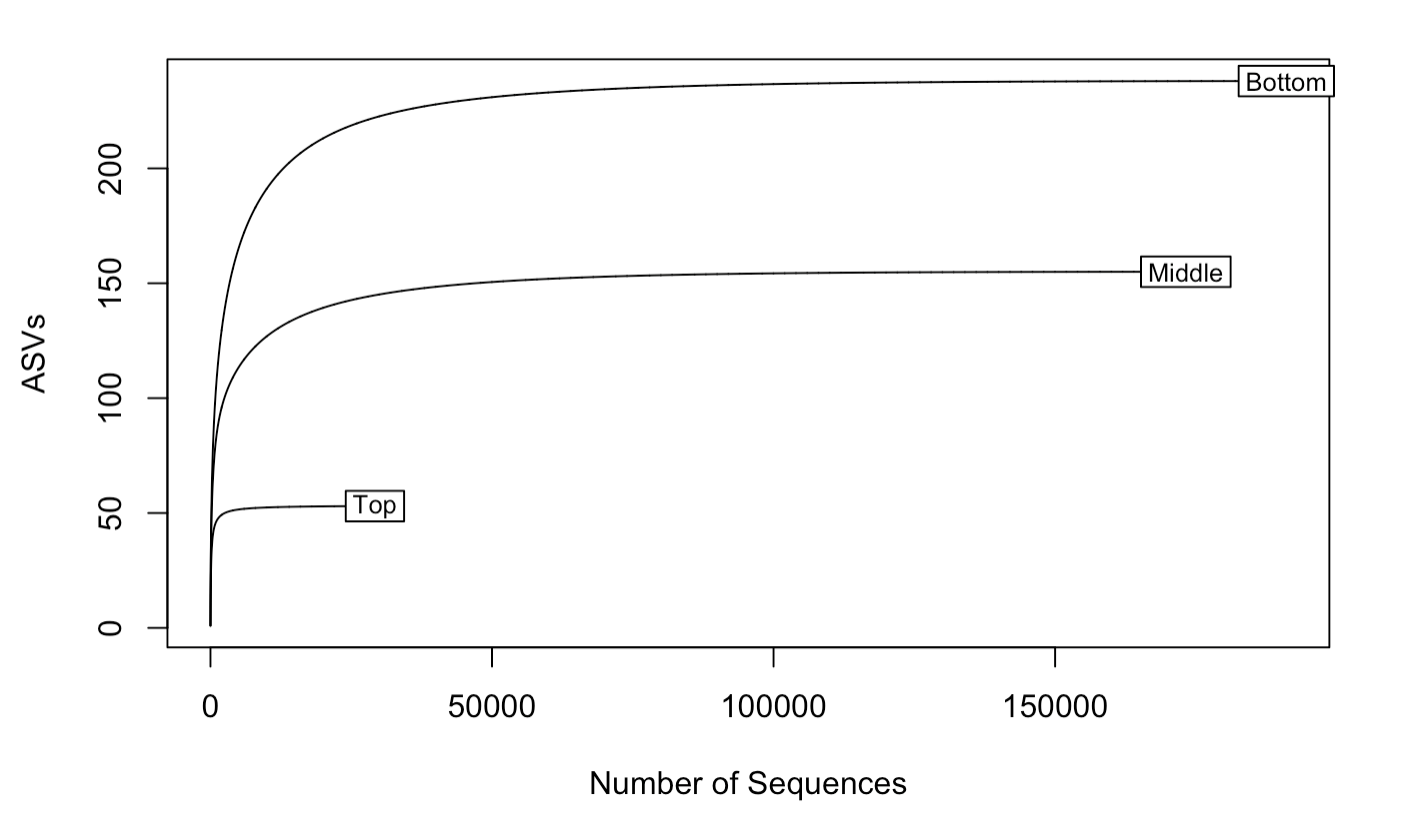 |
| --- |
| **Supplementary Fig. S3.** Rarefaction curve of 16S rRNA gene sequence variants from the Top, Middle and Bottom canopy needle samples. Curves were generated using the sum of each ASV across all needles in each group. |

| 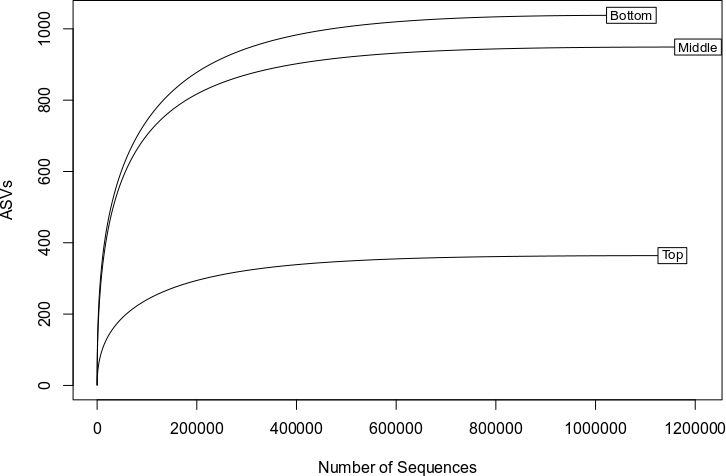 |
| --- |

| **Supplementary Fig. S4.** Rarefaction curve of fungal ITS gene sequence variants from the Top, Middle and Bottom canopy needle samples. Curves were generated using the sum of each ASV across all needles in each group. |
| --- |

**Supplementary Fig. S5.** Multidimensional scaling ordination plot (nMDS) projecting distances in bacterial community similarity (increased similarity = closer grouping) of microbiomes from Top, Middle, and Bottom parts of the canopy. Given the huge separation (dissimilarity in community type) between needles collected from the Top of the canopy and those from the other parts of the canopy, the Bottom and Middle samples have collapsed to a single point; i.e. the changes in community among these samples are trivial when compared with overall difference in the microbiome present in the Top canopy needle samples For this reason, only the distances between the Bottom and Middle samples are show in Figure 2E in the main text.

**Supplementary Fig. S6.** Relative abundance class level taxonomy for bacterial 16S rRNA gene sequence classification. Differentiated to tree height with Top, Middle and Bottom of the tree represented. Taxa with relative abundance <0.5% of ASV’s were excluded from the graph. All other groups include: Deltaproteobacteria, Betaproteobacteria, Proteobacteria, Candidate division WPS-1, Gammaproteobacteria, Actinobacteria, Chlamydia, Bacilli, Flavobacteria, Spartobacteria, Planctomycetia, Candidate Division WPS-2, Armatimodia, Clostridia, and Sphingobacteria.

**Supplementary Fig. S7.** Relative abundance class level taxonomy for fungal ITS gene sequence classification. Differentiated to tree height with Top, Middle and Bottom of the tree represented. Taxa with relative abundance <0.5% of ASV’s were excluded from graph. All other groups include: Cystobasidiomycetes, Taphrinomycetes, Pucciniomycetes, Basidiomycota, Lecanoromycetes, Saccharomycetes, Microbotryomycetes, Orbiliomycetes, GS18, Agaricostilbomycetes, Mortierellomycetes, Archaeorhizomycetes, Pezizomycetes, Spiculogloeomycetes, Umbelopsidomycetes, Rozellomycota, and Archaeosporomycetes.

Supplementary Tables

**Supplementary Table S1. Chemical analysis of foliar needles (mass collection 100 needles) from each respective height of the tree. Conditional (arbitrary) formatting has been used to demonstrate differences between the samples; yellow = lower values and green = higher values.**

|  | N  (%) | C  (%) | Mn | Cu | Ca | B | Fe | K | Mg | Na | P | Zn | Al |
| --- | --- | --- | --- | --- | --- | --- | --- | --- | --- | --- | --- | --- | --- |
|  |  |  | (mg/kg) | (mg/kg) | (mg/kg) | (mg/kg) | (mg/kg) | (mg/kg) | (mg/kg) | (mg/kg) | (mg/kg) | (mg/kg) | (mg/kg) |
| **Top needles** | 1.52 | 50.5 | 313 | 9.7 | 3177 | 15 | 36.6 | 9854 | 1157 | 108 | 2581 | 56.3 | 299 |
| **Middle needles** | 1.06 | 51.2 | 334 | 21.5 | 3864 | 7.6 | 32.9 | 6376 | 676 | 72 | 1236 | 49.2 | 490 |
| **Bottom needles** | 1.05 | 51 | 364 | 17 | 3719 | 10.1 | 27.6 | 6646 | 824 | 77 | 1403 | 57 | 463 |

**Supplementary Table S2. Chao1 richness estimates of bacterial and fungal ASV richness across the phyllosphere microbiome treatment groups. When main-treatment effects were present (p <0.05), pair-wise testing within groups was conducted.**

|  | **Bacteria** | **Fungi** |
| --- | --- | --- |
|  | **P** | **P** |
| **Canopy height** | **<0.0001****** | **<0.0001****** |
| ***Top vs Middle*** | ***<0.0001*******  ***<0.0001*******  ***0.0241****  **0.8522**  **0.4765**  **0.9764** | ***<0.0001*******  ***<0.0001*******  ***0.0167****  **0.2579**  **0.2686**  **0.6225** |
| ***Top vs Bottom*** |  |  |
| ***Middle vs Bottom*** |  |  |
| **Compartment^1^** |  |  |
| **Cardinal points** |  |  |
| **Age** |  |  |

**^1^Compartment refers to endophyte only (*sensu* surface-sterilised) and complete (non-sterilised) needle treatments.**
